# A Case of Kappa Light-Chain Multiple Myeloma With Auer Rod-Like Inclusions and Snapper-Schneid Granules

**DOI:** 10.7759/cureus.83170

**Published:** 2025-04-29

**Authors:** Ant Uzay, Ozgur Ozkayar, Iffet B Gokmen, Can Boynukara, Yildiz Okuturlar

**Affiliations:** 1 Department of Hematology, Acibadem University, Istanbul, TUR; 2 Department of Pathology, Acibadem University, Istanbul, TUR; 3 Department of Internal Medicine, Acibadem University, Istanbul, TUR

**Keywords:** auer-rod, intracytoplasmic inclusions, malignant hematology, multiple myeloma, snapper-schneid

## Abstract

In multiple myeloma, plasma cells can display intracellular and intranuclear inclusions. This case report highlights the coexistence of basophilic Snapper-Schneid granules and Auer rod-like cytoplasmic inclusions. A 73-year-old female patient, with a history of hyperlipidemia, rheumatoid arthritis, and hypertension, presented with swelling in both upper extremities. Subsequent evaluations revealed significant proteinuria, hypercalcemia, and atypical plasma cells characteristic of multiple myeloma, with both Auer rod-like inclusions and Snapper-Schneid granules observed. Auer rod-like inclusion bodies are particularly observed in patients with kappa light-chain-type multiple myeloma. The positivity of these bodies for alpha-N-esterase, acid phosphatase, and beta-glucuronidase suggests they are of lysosomal origin. However, the prognostic implications of Auer rod-like inclusions and Snapper-Schneid granules in multiple myeloma remain unclear. Snapper-Schneid granules, described as oval basophilic granules, are associated with immunoglobulin but are not found exclusively in multiple myeloma.

## Introduction

A number of morphological changes in plasma cells have been reported in multiple myeloma. It is well known that plasma cells can contain intracytoplasmic and intranuclear inclusions, such as Russell and Dutcher bodies. Besides Russell bodies, other cytoplasmic formations can be found in multiple myelomas, such as crystalline inclusions formed by depositions of immunoglobulins or light chains [1]. Apart from these commonly encountered changes, several cases are reported with basophilic staining dense granules called Snapper-Schneid granules and, in rare cases, Auer rod-like inclusions in plasma cell pathologies. We present a case of kappa light-chain multiple myeloma and systemic amyloidosis with multiple azurophilic Snapper-Schneid granules and Auer rod-like cytoplasmic inclusions. Kappa light-chain multiple myeloma is a subtype of multiple myeloma where the malignant plasma cells produce excess kappa light chains without the corresponding heavy chains. It is characterized by the presence of free kappa light chains in the blood or urine.

## Case presentation

A 73-year-old woman with a history of hypertension, hyperlipidemia, and rheumatoid arthritis (RA) presented with complaints of swelling in both her upper extremities, being more prominent on her right arm, with newly added accompanying redness and generalized oedema. Complaints of mild dyspnea and swelling had been present for a few months, and she also reported recurrent episodes of nephrolithiasis. She had a history of hydroxychloroquine, prednisolone, short-term methotrexate, and leflunomide use, which were halted due to the elevation of liver enzymes 6 months prior to admission. On physical examination, +2 pitting oedema and bilateral fine crackles in basal lung sounds with auscultation were noted. A complete blood count showed microcytic anaemia: white blood cells: 7.74 x10^3/uL, hemoglobin: 9.2 g/dL, mean corpuscular volume (MCV): 84.6 fL, thrombocytes: 186x10^3/uL (Table 1). Other lab investigations revealed serum creatinine as 1.06 mg/dL, showing an increase in the last month compared to previous levels of 0.70 mg/dL, blood urea nitrogen (BUN): 15 mg/dL, lactate dehydrogenase (LDH): 213 IU/L, albumin corrected calcium: 10.68 mg/dL, total serum protein: 6.3 g/dL, serum albumin: 2.9 g/dL, erythrocyte sedimentation rate (ESR): 53 mm/hour (Table 1). During the investigation for generalised oedema, proteinuria of 526 mg/24 h was spotted. Urine immunofixation electrophoresis revealed a markedly increased level of kappa light chain: 517 mg/L, lambda light chain: 56.6 mg/L, with a kappa/lambda ratio of 9.13 (Table 1). Serum IgA: 186 mg/dL, IgG: 708 mg/dL, IgM: 254 mg/dL, kappa light chain: 2.22 g/L, lambda light chain: 1.03 g/dL were in normal ranges (Table 1). Serum immunofixation electrophoresis later revealed a monoclonal increase at the kappa band. Serum protein electrophoresis revealed a marked increase of free kappa light chain: 754 mg/L, free lambda light chain: 56.6 mg/dL, with a kappa/lambda ratio of 13.3 (Table 1). Serum beta-2 microglobulin was 4.6 mg/L (Table 1).

**Table 1:** Laboratory tests

Laboratory Test	Laboratory Values	Reference Interval
Leukocyte Number	7.74 x10^3^/uL	4.06-10.6 x10^3^/uL
Haemoglobin	9.2 g/dL	13.0-18.0 g/dL
Mean Corpuscular Volume	84.6 fL	80.0-100.0 fL
Thrombocyte Number	186 x10^3^/uL	150-439 10^3^/uL
Serum Creatinine	1.06 mg/dL	0.7-1.3 mg/dL
Blood Urea Nitrogen	15 mg/dL	6-20 mg/dL
Lactate Dehydrogenase	213 IU/L	120-246 IU/L
Serum Total Protein	6.3 g/dL	5.7-8.2 g/dL
Serum Calcium	10.68 mg/dL	8.7-10.4 mg/dL
Serum Albumin	2.9 g/dL	3.5-5.2 g/dL
Erythrocyte Sedimentation Rate	53 mm/hour	<20 mm/sa
Urine-Lambda Light Chain	56.6 mg/L	0-11.3 mg/L
Urine-Kappa Light Chain	517 mg/L	0-25.8 mg/L
Urine-Kappa/Lambda Ratio	9.13	1.40- 6.20
Serum-Lambda Light Chain	1.03 g/dL	0.9-2.1 g/L
Serum-Kappa Light Chain	2.22 g/L	1.7-3.7 g/L
Serum-Free Lambda Chain	56.6 mg/dL	8.3-27 mg/L
Serum-Free Kappa Chain	754 mg/L	6.7-22.4 mg/L
Serum-Free-Kappa/Lambda Ratio	13.3	1.35-2.65
Immunoglobulin A	186 mg/dL	70-400 mg/dL
Immunoglobulin M	254 mg/dL	50-300 mg/dl
Immunoglobulin G	708 mg/dL	650-1600 mg/dl
Serum B2 Microglobulin	4.6 mg/L	1.09-2.53 mg/L

**Figure 1:**
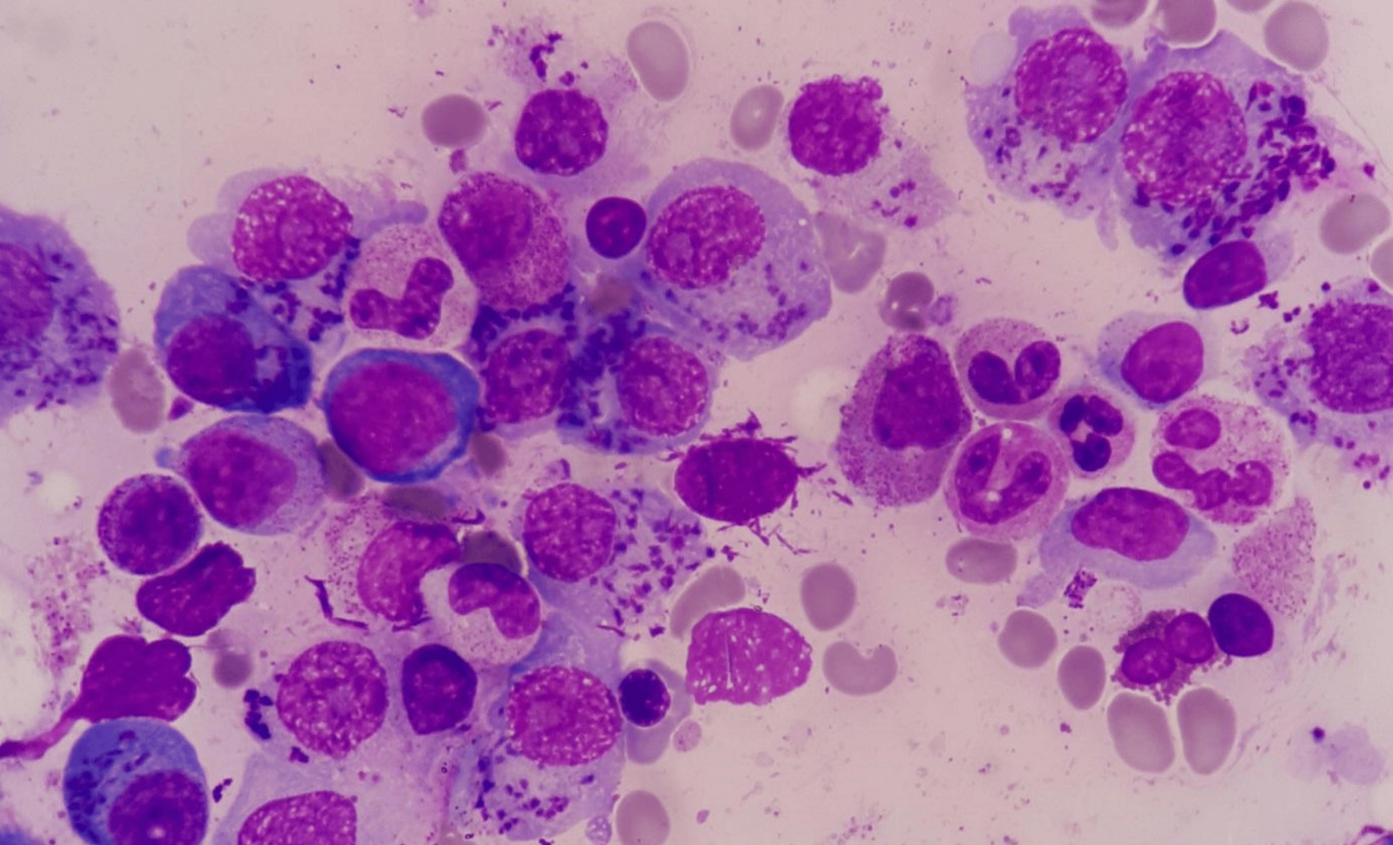
Multiple intracytoplasmic inclusion bodies, coarse azurophilic Snapper-Schneid granules in atypical plasma cells of multiple myeloma. Bone marrow smear, stained with Giemsa (x100)

**Figure 2:**
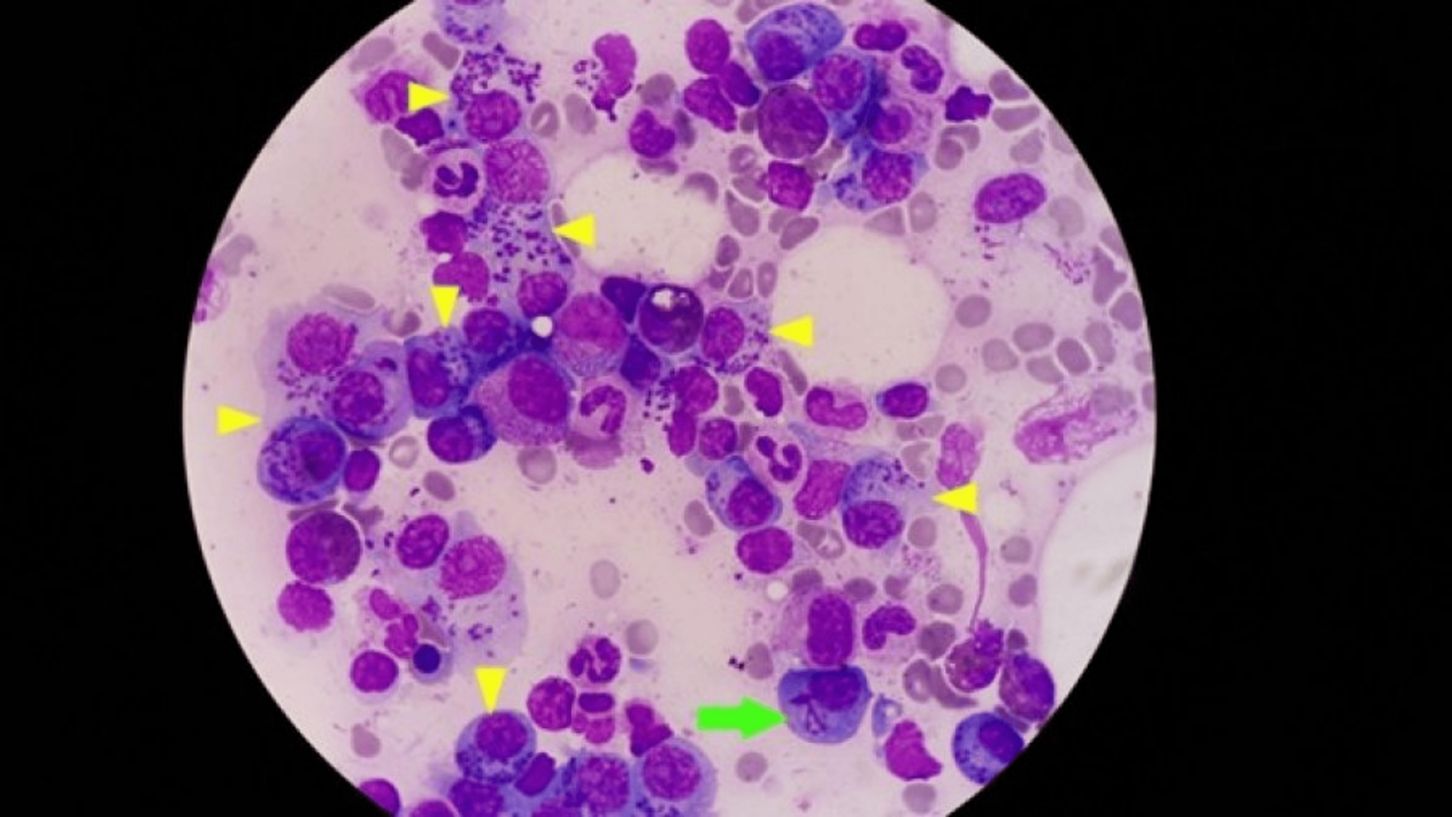
Snapper-Schneid granules (yellow arrowheads) and needle-shaped Auer rod-like inclusions of multiple myeloma cells in a single field of view (green arrows) bone marrow smear, stained with Giemsa (x100)

**Figure 3:**
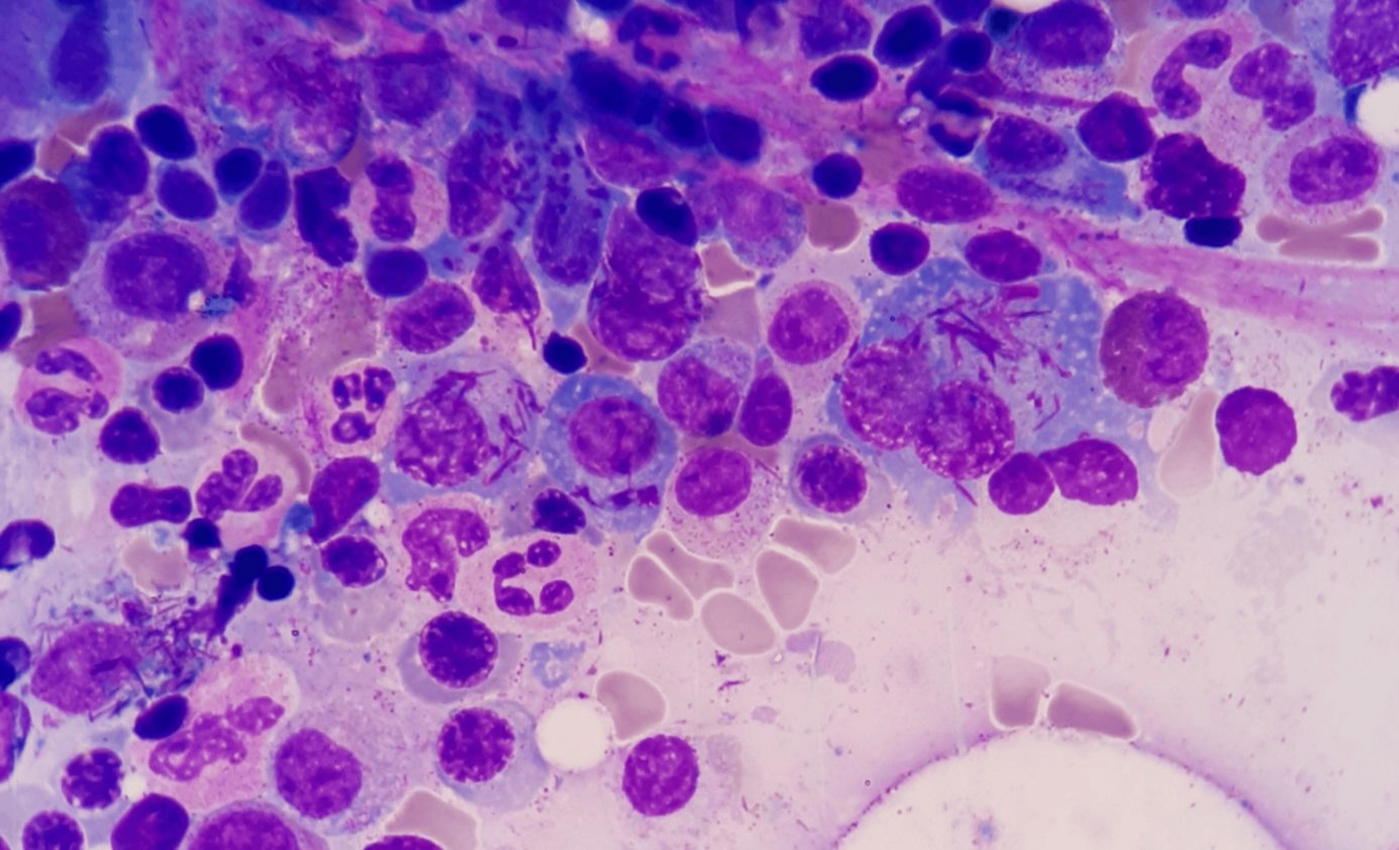
Multiple needle-shaped intracytoplasmic inclusion bodies seen in atypical plasma cells of multiple myeloma, resembling Auer rods of acute promyelocytic leukemia. Bone marrow smear, stained with Giemsa (x100)

**Figure 4:**
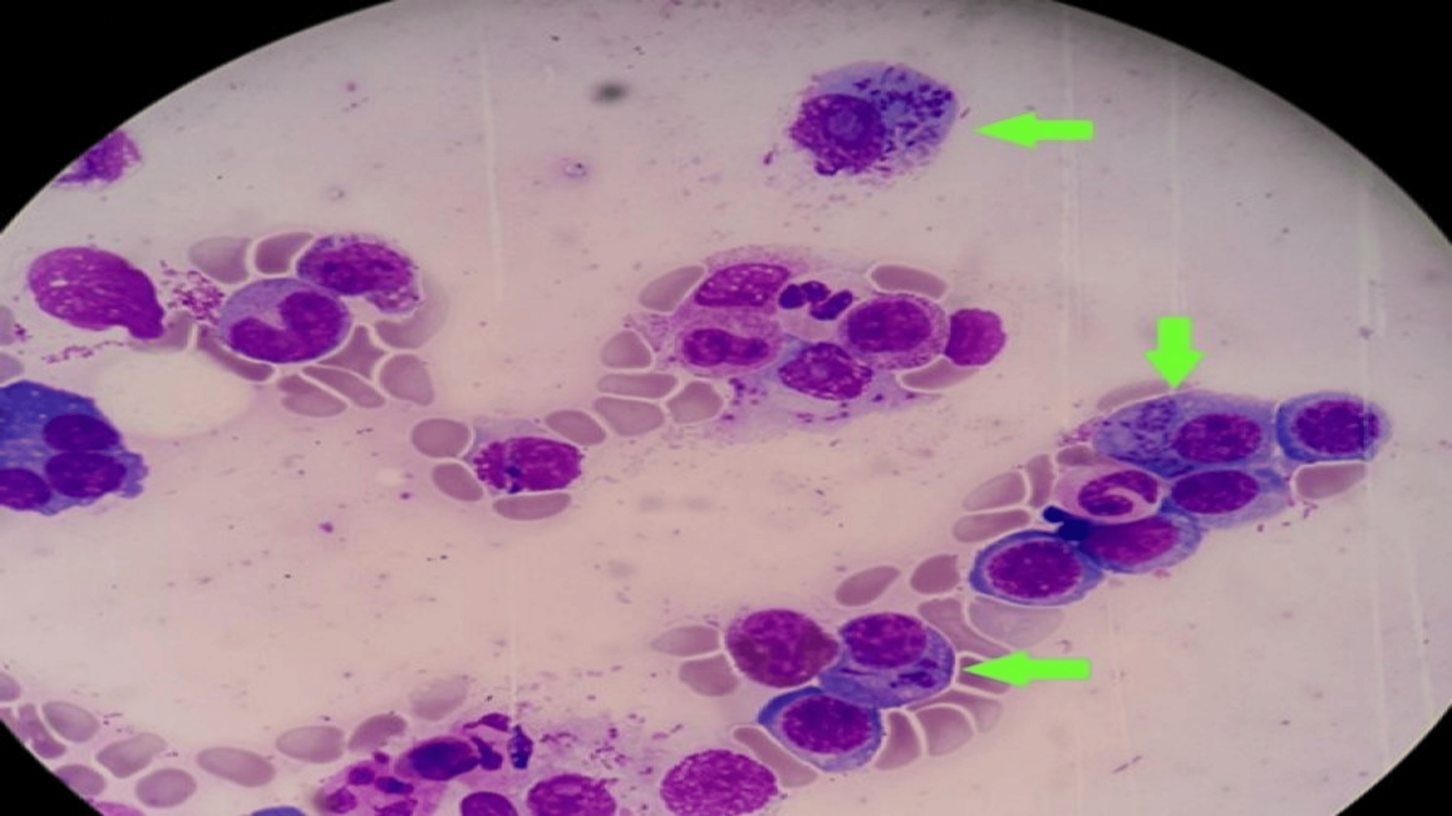
Snapper-Schneid granules and needle-shaped Auer rod-like inclusions seen coexisting in a single plasma cell (green arrows) bone marrow smear, stained with Giemsa (x100)

No lytic bone lesions were found in the skeletal survey. Bone marrow aspiration, biopsy, and smear were done with the preliminary diagnosis of multiple myeloma and suspected systemic amyloidosis. Myelogram reported hypercellularity, atypical plasma cells making up approximately 17% of all nucleated cells. Maturation stages of all three series showed no interruption, and no other series of cells were found to be proliferative or infiltrative. Giemsa-stained plasma cells showed an atypical presentation. Multiple coarse intracytoplasmic azurophilic Snapper-Schneid granules (Figures 1, 2) were visualized in the majority of the monoclonal plasma cells. Lesser plasma cells had bundles of multiple intracytoplasmic needle-shaped inclusions resembling Auer rods of acute promyelocytic leukaemia (Figures 2, 3). Also, these two findings were found to exist concurrently in a single cell (Figure 4).

**Figure 5:**
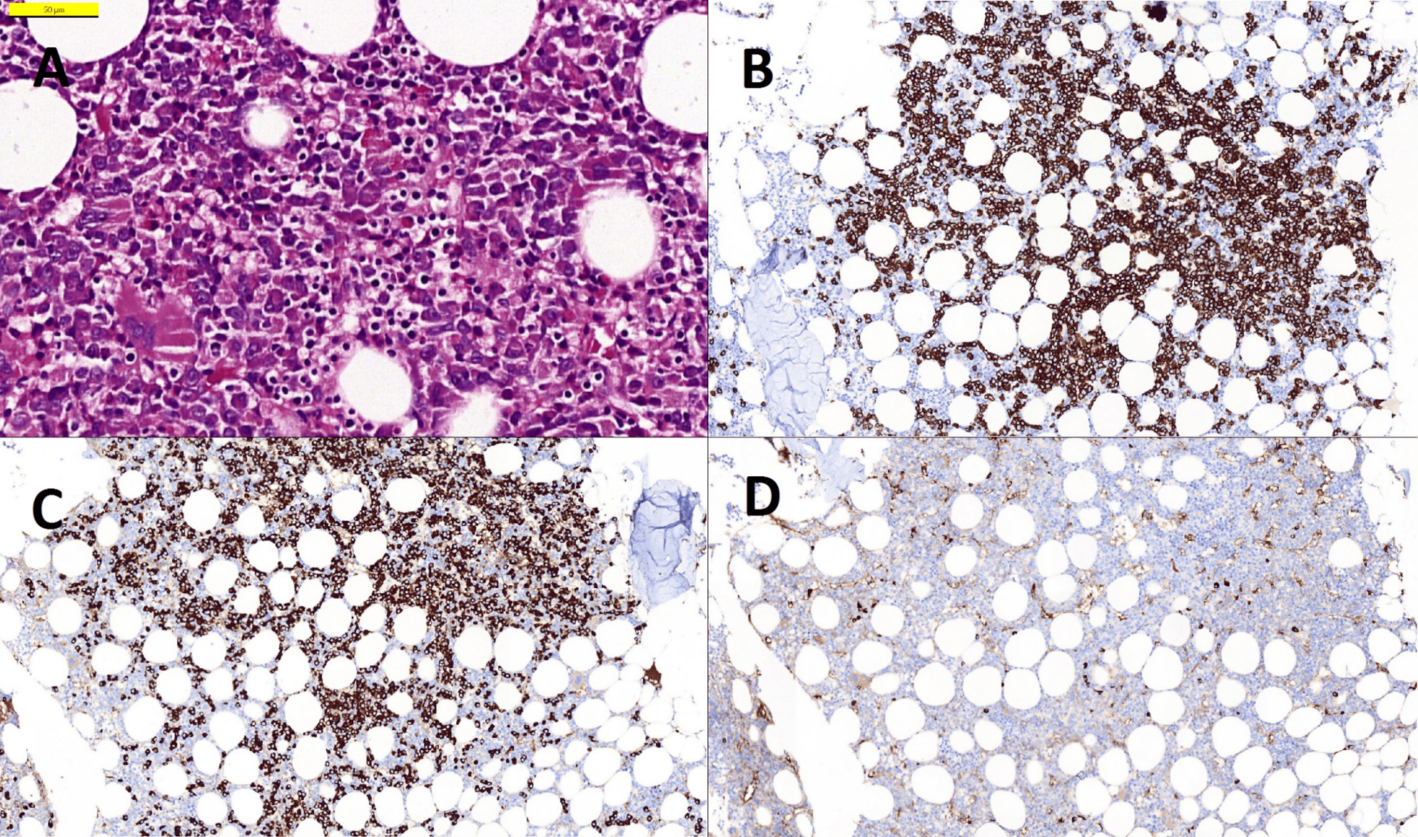
Immunohistochemistry A: H&E-stained bone marrow biopsy sections show clusters of atypical plasma cells with central nucleolus (400x); B: Immunohistochemical staining with CD138 antibody highlights large clusters of plasma cells (100x); C and D: Kappa, Lambda: 99% of plasma cells are positive with Kappa antibody, Lambda is positive only in a few cells at the same area (100x).

**Figure 6:**
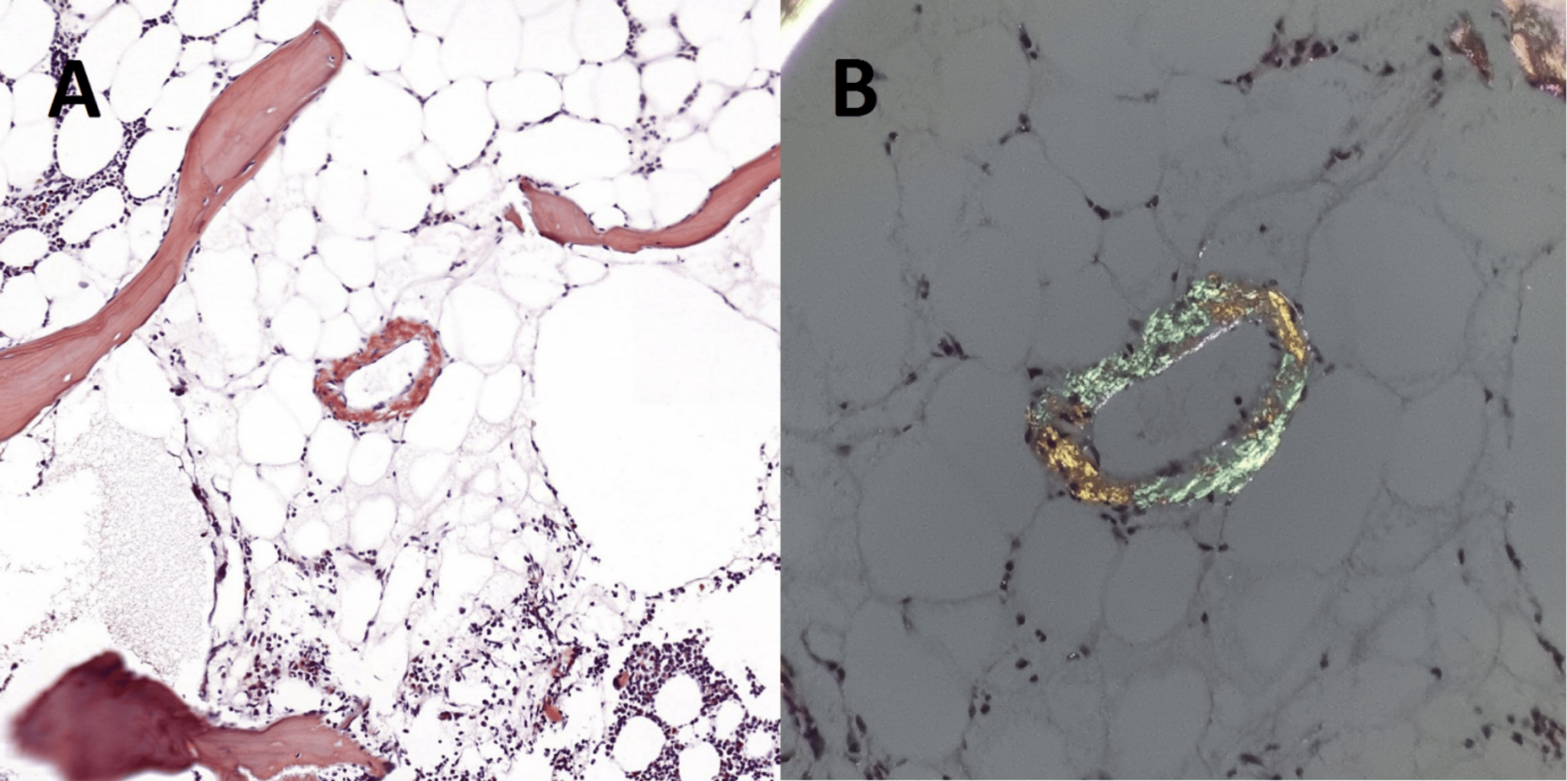
Staining with Congo red shows positivity in small vessels (200x) (A); this deposition is verified by apple-green birefringence under polarized light (B).

Bone marrow aspiration biopsy confirmed the presence of CD138+ and CD38+ atypical plasma cells, making up approximately 20% of all nucleated cells. Cellularity showed heterogeneity in areas between 15% and 65%. The mean cell-to-fat ratio was 45/55. Immunohistochemically, 99% of all plasma cells were found to be kappa positive (Figure 5). Amyloid deposition was detected in two different areas of vascular and connective tissue in the bone marrow stroma (Figure 6). The bone marrow biopsy findings were compatible with multiple myeloma and amyloidosis. Multiple myeloma genetic panels were studied using Fluorescent Insitu Hybridisation (FISH) analysis, which detected normal cytogenetic results. The FISH panel included IGH/FGFR3, IGH/CCND1, IGH/CCND3, IGH/MAF, IGF/MAFB, IGH, 13q14 RB1, del D134S319, trisomy 12, 17p13.1 CKS1B/CDKN2C, and MYC. The patient has been under the standard myeloma treatment for 3 years.

## Discussion

The first report of Auer rod-like crystalline structures in multiple myeloma cells dates back to 1940, according to Steinmann’s description [2]. A few years later, I. Snapper and B. Schneid reported a case where they visualized Auer-rod-like inclusions and first ever described dense basophilic staining granules in plasma cells as in a multiple myeloma patient after treatment with stilbamidine [3]. Since then, various studies have been conducted to understand their nature and importance in prognosis. A literature review in 2007 revealed that Auer rod-like inclusions are exclusively found in myeloma with kappa-type paraprotein, as with our patient [1]. Immunocytochemical studies revealed no reaction with antibodies against immunoglobulins, light chains, and amyloid A antibodies inside the inclusions [4]. The key findings in these studies are alpha-N-esterase, acid phosphatase, and beta-glucuronidase positivity, which is suggestive of lysosomal origin [4-7]. Due to its probable lysosomal origin, there may be a link to lysosomal dysfunction. In five different reported cases, adult Fanconi syndrome was found as a concomitant illness. There is still no certainty over their relationship [1]. Snapper-Schneid granules are ovoid-shaped basophilic inclusions that represent precipitated immunoglobulin [8]. Individual case reports have proposed that Snapper-Schneid granules may not be restricted to myeloma cases; they also may be seen in reactive plasmacytosis [9]. It is still unknown whether these inclusions are of any prognostic significance [10]. The clinical significance remains unclear, underscoring the necessity for further investigative studies to elucidate its implications.

## Conclusions

Auer rod-like inclusion bodies are mainly observed in kappa light-chain type multiple myeloma patients. The positivity for alpha-N-esterase, acid phosphatase, and beta-glucuronidase suggests that these bodies are of lysosomal origin. The impact of Auer rod-like inclusions and Snapper-Schneid bodies on prognosis in multiple myeloma is not fully understood. The clinical relevance of this finding remains uncertain, emphasising the need for further research to clarify its implications.
